# Continuing Professional Development—Radiation Therapy

**DOI:** 10.1002/jmrs.70136

**Published:** 2026-10-09

Maximise your continuing professional development (CPD) by reading the selected article and answering the five questions. Please remember to self‐claim your CPD and retain your supporting evidence. Answers will be available via the QR code and published in JMRS—Volume 74, Issue 4, December 2027.

## 3D Printing a Clinically Available Solution From Plaster‐Based Positives in Superficial Radiation Therapy

Rory Hartley, Linda Bell, Toby Lowe, Jeremy Booth, Sarah Bergamin, Dasantha Jayamanne, Ryan Brown, https://doi.org/10.1002/jmrs.70098.

1. What was the primary aim of the study?
  - To compare skin cancer treatments
  - To measure radiation dose during treatment
  - To test new radiation therapy machines
  - To compare three methods for creating facial lead shielding
2. Which method produced the most accurate 3D facial model?
  - Plaster Cast Positive Mould
  - Optical Scan 3D Print (OS3DP)
  - CT Scanned 3D Print (CT3DP)
  - The methods demonstrated similar accuracy
3. A radiation therapy department is considering an alternative method for producing facial lead shielding. Which CT3DP advantage would support its use?
  - It eliminates the need for imaging
  - It provides greater accuracy and reproducibility
  - It requires no post‐processing
  - It has the lowest equipment costs
4. A department is comparing the CT3DP and OS3DP workflows. Which consideration is relevant when assessing their suitability for clinical use?
  - Both workflows require approximately 2 h
  - OS3DP takes substantially longer than CT3DP
  - Both workflows take approximately 6 h to complete
  - CT3DP requires approximately 10 h to complete
5. A radiation therapy department is considering CT3DP for facial lead shielding. Which factors should be considered when applying the study findings to practice?
  - Accuracy, reproducibility, workflow and clinical context
  - Accuracy and reproducibility only
  - Workflow time only
  - Participant preference only

## Answers

Scan this QR code or www.surveymonkey.com/r/JMRS_Dec2026_RT to find the answers.

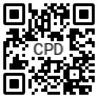
